# A Community-Based Model of Care During the Fourth Wave of the COVID-19 Outbreak in Ho Chi Minh City, Vietnam

**DOI:** 10.3389/frai.2022.831841

**Published:** 2022-04-04

**Authors:** Lan N. Vuong, Nghia Huynh, Dat Q. Ngo, Vinh N. Nguyen, Khoa D. Duong, Nguyen N. Tran, Truyen P. Le, Nghia A. Nguyen, Thao T. P. Doan, Duy L. Pham, Tu H. K. Trinh, Quan T. T. Vu, Phong H. Nguyen, Tuan D. Tran

**Affiliations:** University of Medicine and Pharmacy at Ho Chi Minh City, Ho Chi Minh City, Vietnam

**Keywords:** COVID-19, SARS-CoV-2, pandemic, community, Vietnam

## Abstract

In response to a call for help during a surge in coronavirus disease-19 (COVID-19) cases in Ho Chi Minh City in July 2021, the University of Medicine and Pharmacy at Ho Chi Minh City developed and implemented a community care model for the management of patients with COVID-19. This was based on three main principles: home care; providing monitoring and care at a distance; and providing timely emergency care if needed. One team supported patients at home with frequent contacts and remote monitoring, while a second team transferred and cared for patients requiring treatment at field emergency care facilities. COVID-19-related mortality rates at the two districts where this approach was implemented (0.43% and 0.57%) were substantially lower than the overall rate in Ho Chi Minh City over the same period (4.95%). Thus, utilization of a community care model can increase the number of patients with COVID-19 who can be effectively managed from home, and use of field emergency care facilities limited the number of patients that had to be referred for tertiary care. Importantly, the community care model also markedly reduced the mortality rate compared with traditional methods of COVID-19 patient management.

## Introduction

Despite the rollout of vaccinations worldwide, many regions continue to experience new waves of coronavirus 2019 (COVID-19) disease. Contributing factors include suboptimal vaccination rates and the emergence of new variants of SARS-CoV-2, especially the highly transmissible delta variant (Shiehzadegan et al., 2021). With up to 15% of those infected with the virus requiring hospitalization (Docherty et al., 2020), demand for hospital care, including intensive care unit (ICU) admission can overwhelm capacity. This can result in avoidable deaths (Wood et al., 2020). In addition, management of high numbers of severely ill patients with COVID-19 has been shown to be independently associated with higher in-hospital COVID-19-related mortality (Soria et al., 2021). These factors provide a sound and urgent rationale for the development of alternative and creative care pathways that help to prevent hospitals becoming overwhelmed.

Nacoti et al. (2021) proposed a three-tier community-based model for the management of adult patients with COVID-19 to avoid hospital overcrowding and identify the most appropriate levels of assistance and locations of care. This advocated home care for patients with a mild COVID-19 (oxygen saturation [SpO_2_] ≥90%), community care center treatment for those with severe disease (SpO_2_ <90%), and hospital care for critical cases (Nacoti et al., 2021).

Such an approach clearly has merit, but the article by Nacoti et al. (2021) did not include any data showing the effects of applying their community-based model on outcomes for COVID-19 patients. However, our recent experience in Vietnam provides support for a community care model for the management of COVID-19 in terms of reducing the impact of a high infection rate on hospitals and hospital capacity, and with respect to patient outcomes.

## Fourth Wave of Covid-19 in Vietnam

After very few cases and deaths during initial waves of COVID-19 infection in Vietnam, new cases began to increase dramatically at the end of May 2021 (World Health Organization, 2021). Ho Chi Minh City was particularly hard hit, with 8,000–9,000 cases per day. One of the worst affected areas was District 10, which has an area of 5.72 km^2^ and a population of 234,819. Within this district, the number of COVID-19 cases peaked on 25th July (617 new cases). Overall, 81% of cases had mild disease, 14% had severe disease, and 5% were critically ill (Wu and McGoogan, 2020).

In response to the growing case numbers, hundreds of community centers and temporary hospitals were built in Ho Chi Minh City, but the number of cases exceeded the capacity of these community centers and temporary hospitals, and also the critical care hospitals. Therefore, the healthcare system was overwhelmed. In addition, healthcare professionals working in the community centers and temporary hospitals often became infected with COVID-19.

Although the majority of cases were of mild severity, COVID-19 patients who were isolating at home were worried about not receiving timely care from healthcare professionals and about there not being enough hospital capacity to treat them if they became severely ill. Some patients who had only mild symptoms were so worried that they went to the hospital anyway, which meant that the hospitals became even more overwhelmed.

## Community Care Model

**Figure 1:**
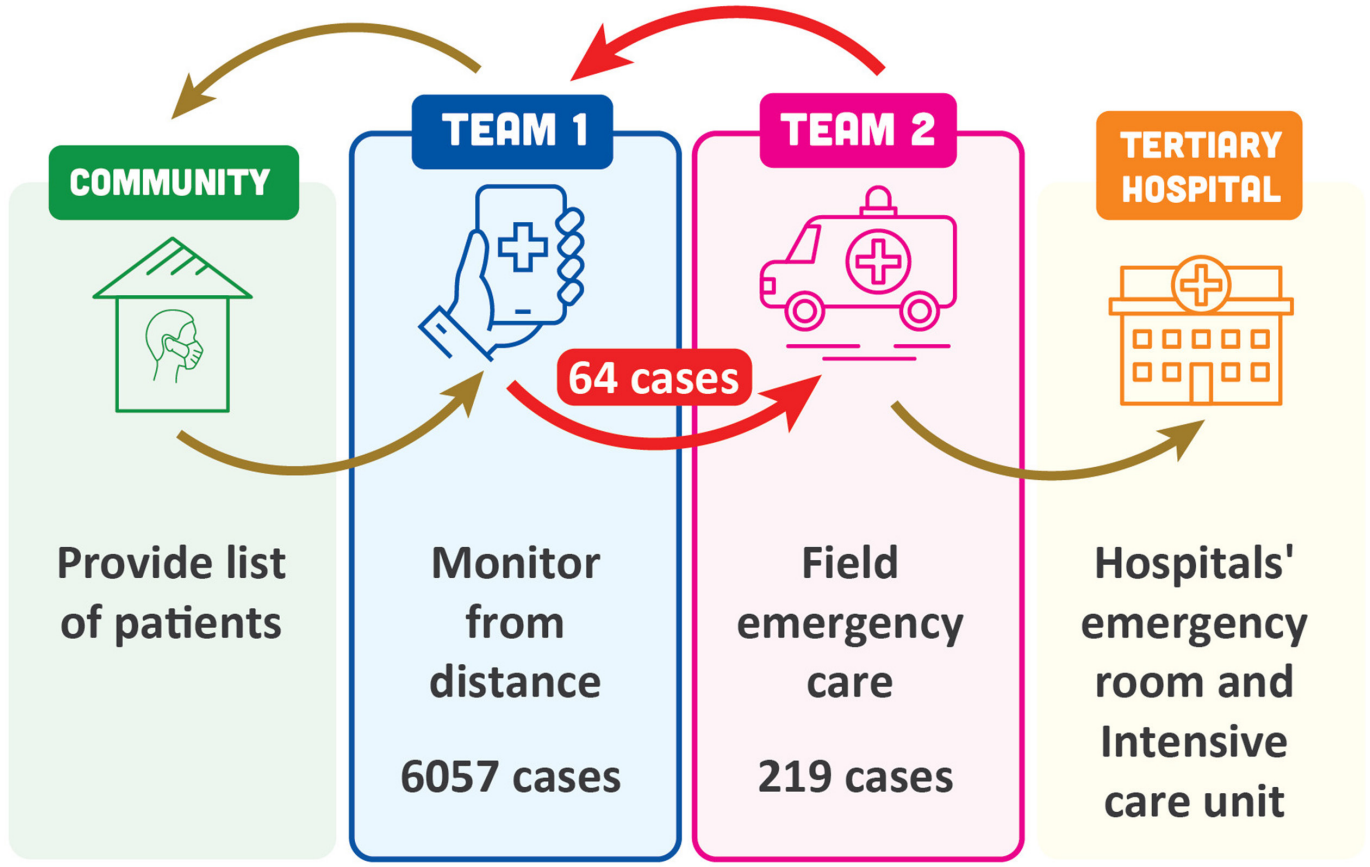
Community-based care model for patients with COVID-19 in District 10, Ho Chi Minh City, Vietnam.

The University of Medicine and Pharmacy at Ho Chi Minh City (UMP) was asked to help with the COVID-19 response in District 10. The rapid increase in case numbers and disease spread meant that the transfer of new cases to community centers was not feasible. Therefore, a new community care model was applied for the care of COVID-19 patients in this district (Figure 1). The objectives of this approach were to reduce the death rate in patients infected with SARS-CoV-2 and avoid health facilities becoming overloaded. There were three main principles:

- Home care for COVID-19 patients.
- Provide monitoring and care for COVID-19 patients at distance.
- Provide timely emergency care.

The model organized healthcare professionals into two teams. Team 1 took care of patients at home using an online platform (e.g., online audio or video call). Patients were contacted daily, and information obtained on oxygen saturation (determined using personal units supplied to the patients), respiratory rate and signs of respiratory distress. This allowed classification of patients as being at high, average or low risk of progressing to severe disease, and allowed early detection of clinical deterioration. Frequency of contact from the medical team was based on the risk classification. Patients could also contact the medical team at any time. Treatments available to be provided to patients at home included oxygen, corticosteroids and anticoagulants, and treatment was individualized for each patient. Team 1 included 62 UMP faculty members, ten resident doctors, five other postgraduates, and 263 undergraduates.

Team 2 set up and provided field emergency care. The field emergency care center had 20 beds. This team was contacted by members of Team 1 when they determined that a patient at home needed checking and respiratory assistance. Team 2 went to the patient's house, picked them up and transferred them to the field emergency care facility. Care provided in this facility included continuous positive airway pressure, remdesivir, corticosteroids, anticoagulants, prone positioning and physical therapy. If patients were stable and doing well after emergency care they were sent home to resume care from Team 1. Alternatively, if severe disease was present, patients were transferred for higher level care (temporary hospital or critical care hospital). Team 2 included twelve UMP faculty members (two medicine, ten nursing), eleven resident doctors, and 26 students (fourteen medical, eleven nursing and one pharmacy).

### Impact of Community Care

The total number of cases in District 10 managed using this community care model between 27 July and 10 September 2021 was 6,057. Sixty-two cases had emergency care at home without transfer to the field emergency care facility, and 64 cases were transferred for emergency care in the field. In addition to these 64 cases referred by Team 1, another 155 cases were also admitted to the field emergency facility (*n* = 219 in total). Of these, 58% were female, 42% were male, and although all age groups from 18 to >80 years were represented, the majority (157/219; 72%) were aged >50 years. A high proportion of patients requiring additional care (71%) had comorbidities, including hypertension (32%), diabetes (25%), cardiovascular disease (12%), metabolic disease (10%) or other disorders (18%); 31% of patients had more than one comorbidity. The majority of Team 1 patients transferred to Team 2 went on to need tertiary care (58/64; 91%) (Table 1). The overall mortality rate for patients fully managed using the community model was 0.43% (Table 1). This rate is substantially lower than the overall COVID-19-related death rate in Ho Chi Minh City, which has been estimated at 4.95% (Nikkei Asia, 2021).

**Table 1:** Summary of treated patient numbers and outcomes.

**Number of** **patients**	**Monitored by** **Team 1**	**Transfer to** **Team 2**	**Recover and** **discharge from** **Team 2 care**	**Transfer to tertiary hospitals**			**Mortality rate** **(of monitored cases)**
				**Total**	**Recovery**	**Death**	
District 10	6,057	64	6	58	32	26	0.43%
District 8	8,188	250	100	150	103	47	0.57%

When daily case numbers in Ho Chi Minh City were peaking in early August (504 cases on 4th August), UMP was contacted to expand the community care model to District 8, which has an area of 19 km^2^ and a population of ~451,290.

A total of 8,188 cases were managed by Team 1, who referred 250 cases to Team 2 for admission to the field emergency facility. Similar to District 10, the majority of patients cared for by Team 2 in the field emergency facility were female (57%) and aged ≥50 years. Again, a high proportion of patients requiring additional care had comorbidities, including hypertension (51%), diabetes (23%), cardiovascular disease (11%), metabolic disease (10%) or other disorders (12%); 30% of patients had more than one comorbidity. Three-quarters of all patients transferred for emergency care in the field recovered, and the remaining 25% died. The proportion of patients from Team 1 who were transferred to Team 2 who went on to need tertiary care was 60% (150/250) (Table 1). The District 8 mortality rate for patients fully managed using the community model was 0.57% (Table 1), which again was much lower than the estimated 4.95% COVID-19-related death rate in Ho Chi Minh City (Nikkei Asia, 2021).

A good indicator of the positive impact of the community care model in District 8 is shown by the fact that although the number of cases increased from 11,000 to 19,457 between 10th August (when the model was initiated) and 18th September, the number of deaths decreased from 16–29 per day to 1–4 per day, and there were no deaths on some of the days over this period.

### Expanding the Community-Based Care Model

After applying the community-based care model in the two districts described above, we found that model set-up did not require much money, but personnel were an important issue. Therefore, we created a guideline for setting up the community-based model in other districts that wanted to apply our model to treat their patients. As an alternative, we also suggested a way to integrate our model into a local healthcare system.

The following guidelines apply to setting up the community-based model:

For Team 1:

- Create one monitoring group for every 20–30 patients; this should include one doctor or nurse and two medical students.
- Include a group of experts in critical care or respiratory medicine at each district to provide a consultation service for the monitoring group regarding whether or not to transfer patients from home to the field emergency care center because seeing patients through an online platform is not always the easiest way to recognize that they require respiratory assistance.
- Have a clear checklist for checking patients every day and clear criteria for escalating the care of a patient to Team 2.
- Each monitoring group needs one or two smartphones or laptops connecting to WiFi.

For Team 2:

- For every 20 beds in the field emergency care center it is suggested to have at least twelve healthcare providers, including two doctors specialized in critical care or respiratory medicine, four nurses, four medical students, and two paramedics.
- Ensure sufficient supply of medication and oxygen for the primary care of patients with respiratory distress.

Integration of the model into local healthcare system:

- For districts that do not want to set up a new community model, local healthcare systems could be modified to operate in a similar way to the model. It is suggested that the District health center can perform Team 1 tasks and the District hospital can provide the care offered by Team 2. Both the health center and the hospital need to connect closely, as is the case for member of Team 1 and Team 2 in the community model. To achieve this close connection, there needs to be leadership from a steering committee or district government.

## Discussion

The community care model developed and used in two districts of Ho Chi Minh City provided individualized care for patients at home, with escalation of care to field emergency facilities if needed, followed by referral to tertiary care. Regular contact from healthcare professionals made patients with COVID-19 comfortable about staying at home during the course of their disease. Despite the majority of the patients being managed at home, the community care model still provided timely access to emergency care when required, resulting in a lower death rate compared with areas in the same city not using this model. Another important benefit of the remote care used in the community model was a reduction in the number of healthcare providers infected with COVID-19 while caring for mildly symptomatic patients, meaning that these personnel could remain at work. Effective triage of patients means that hospitals were less likely to get overwhelmed, and that the right patients were transferred to the right level of care at the right time.

Community care models such as this are essential to optimize patient management and outcomes during ongoing phases of the pandemic. They can help prevent hospital systems becoming overwhelmed, as has been common during the pandemic (Tangcharoensathien et al., 2021). In addition to the possibility of having to ration care for COVID-19 patients due to bed or equipment shortages (Srinivas et al., 2021), there are several issues associated with significant hospital capacity being required to manage patients with COVID-19. This includes nosocomial infection with SARS-CoV-2 in other patients and healthcare workers (Nguyen et al., 2020; Richterman et al., 2020), and substantial reductions in the provision of ongoing services such as elective surgery and oncology programs (Jazieh et al., 2020; Kuderer et al., 2020; Burden et al., 2021). This impact of COVID-19-related disruptions in healthcare provision has an adverse effect on non-COVID-19-related conditions, which could contribute to potentially avoidable morbidity and mortality associated with non-COVID conditions (Mulholland et al., 2020).

There is also the risk of a subsequent explosion of non-communicable diseases (NCDs) as essential health services, screening programs, and early interventions are disrupted or postponed due to the pandemic. In 2020, the World Health Organization (WHO) reported that disruption to services for hypertension management, treatment of diabetes and its complications, cancer and cardiovascular disease emergencies occurred in 64%, 62%, 54%, and 46% of countries, respectively (World Health Organization, 2021a). Data from the WHO national pulse survey, released in April 2021, showed that 90% of essential health services were disrupted more than one year into the COVID-19 pandemic (World Health Organization, 2021b). Among the services severely affected (disruption reported by >40% of countries) were those for several NCDs, including hypertension, diabetes and mental health (World Health Organization, 2021c). Therefore, there is a clear need to mitigate the impact of COVID-19 infection surges on hospitals and healthcare systems.

Based on our real-world data, utilization of a community care model can increase the number of patients with COVID-19 who can be effectively managed from home, and use of field emergency care facilities limited the number of patients that had to be referred for tertiary care. Most importantly, the COVID-19 mortality rate was substantially lower in patients managed using the community care model compared with usual care. Another advantage to the community model developed by the team at UMP was that, as university faculty members, we were able to involve junior staff and students. This allowed us to teach them important skills, including history taking and caring for patients with mild respiratory disease symptoms. Resident doctors learnt how to recognize respiratory distress and take care of patients with moderate or severe disease patients at the field emergency care site. In addition, implementation of the model demonstrated the value of a multidisciplinary approach to patient care, learning from colleagues, teamwork, and problem-solving skill, all of which will be useful in their future careers.

In summary, we have confirmed the effectiveness and scalability of a community-based care model for the management of COVID-19 during surges in infection case numbers.

## Data Availability Statement

The raw data supporting the conclusions of this article will be made available upon reasonable request to the corresponding author.

## Ethics Statement

Ethical review and approval was not required for the study on human participants in accordance with the local legislation and institutional requirements. Written informed consent for participation was not required for this study in accordance with the national legislation and the institutional requirements.

## Author Contributions

LNV, TDT, NH, and DQN were members of the steering committee for development of the community model. VNN, NAN, TTPD, DLP, THKT, QTTV, and PHN were in charge of Team 1 in both districts. KDD, NNT, and TPL were in charge of Team 2 in both districts. The first draft of manuscript was written by LNV and TDT. All authors were involved in the decision to publish the article and in critical revisions of the manuscript.

## Conflict of Interest

The authors declare that the research was conducted in the absence of any commercial or financial relationships that could be construed as a potential conflict of interest.

## Publisher's Note

All claims expressed in this article are solely those of the authors and do not necessarily represent those of their affiliated organizations, or those of the publisher, the editors and the reviewers. Any product that may be evaluated in this article, or claim that may be made by its manufacturer, is not guaranteed or endorsed by the publisher.
